# Providers’ Perceived Value of Clinical Pharmacist and Technician Services within Primary Care Clinics

**DOI:** 10.3390/pharmacy10060175

**Published:** 2022-12-17

**Authors:** Alan Abbinanti, Stacey Slager, Kyle Turner, Erin Gurney, Golden Benjamin Berrett, Nicholas Cox

**Affiliations:** 1 University of Utah Health, Salt Lake City, UT 84132, USA; 2 Department of Pharmacotherapy, College of Pharmacy, University of Utah, Salt Lake City, UT 84112, USA; 3 Richfield Family Pharmacy, Richfield, UT 84701, USA

**Keywords:** ambulatory care, primary care, practice management, clinical pharmacy, comprehensive mediations management, pharmacy interventions, provider support

## Abstract

Several studies have demonstrated the benefit of clinical pharmacy services in primary care. However, studies are limited on providers’ perceived value of embedded primary care pharmacy teams. The purpose of this project was to determine how primary care clinical pharmacists and technicians provide value to medical providers. Primary care providers in University of Utah health clinics where primary care clinical pharmacists are embedded were invited to participate in one-on-one, semi-structured interviews. Interview sessions were recorded, transcribed, and de-identified. The transcripts were coded and analyzed to determine common themes. Questions were on various topics, including what is of greatest value to them, pharmacy integration into care teams, provider burnout, provider happiness at work, provider workload, and provider retention in the health system. In total, 25 interviews were conducted from nine different clinics (response rates of 19.7% for providers and 81.8% for clinics). Coding revealed themes of increased job satisfaction, enhanced patient care, decreased workload and burnout, and a desire for increased access to clinical pharmacy services. The responses related to clinical pharmacists in primary care were overwhelmingly positive, and providers almost unanimously expressed the need for more pharmacy services in primary care.

## 1. Introduction

University of Utah Health (U of U Health) has adopted the comprehensive medication management (CMM) practice model for use in its pharmacy primary care services (PPCS). CMM is a standardized process that ensures a pharmacist assesses the patients’ medications for an appropriate indication, effectiveness, safety, and any barriers to adherence [1,2]. Through collaborative practice agreements (CPAs) with physicians, PPCS pharmacists are able to manage disease states for patients utilizing their own clinical expertise through the CMM process. The CPAs that PPCS pharmacists utilize in the U of U Health system are broad and allow pharmacists to initiate and adjust medications, order and review labs, and provide patient education and lifestyle management. The PPCS pharmacists employing these CPAs are embedded in the clinic primary care offices, rather than the pharmacies, to better provide true team-based collaborative health care to their patients.

The utilization of CMM and CPAs support the quadruple aim of enhancing patient experiences, improving population health, reducing costs, and improving the work life of health care providers [3]. Several studies have demonstrated the benefits that pharmacy services utilizing CMM provide to patients [4,5,6,7,8,9,10,11,12,13]. However, studies are limited on the perceived value of CMM services provided by embedded pharmacy teams utilizing CPAs. One qualitative study published on the topic demonstrated that providers found that primary care pharmacy services improved their work life [14]. While this study was promising, it did not encompass all potential areas of value that a PPCS pharmacist can provide when they are embedded in a primary care team.

Uncovering a more complete picture of what providers value in their PPCS pharmacy services team is essential for continuing to develop these services and improve on what has already been conducted. This study will add to the literature while providing qualitative information on all aspects of the quadruple aim to improve primary care pharmacy services within the health system and in health systems across the country. Therefore, it was our aim to identify, through individual interviews, how PPCS pharmacist and technician services provide value to medical providers.

## 2. Materials and Methods

This was a qualitative research study designed to better understand what medical providers value most in PPCS pharmacist and technician services through individual semi-structured interviews followed by qualitative coding and theming. The study took place between October 2021 and April 2022, with all provider interviews taking place in January 2022. The study was deemed exempt from review under category two by the University of Utah’s institutional review board.

### 2.1. Recruitment

To be eligible for inclusion in the study, the individual needed to be considered a primary care provider by U of U Health, work in a clinic with an embedded PPCS pharmacist, and have utilized PPCS in their current role. The only exclusion criterion was working in their current role with a PPCS pharmacist for less than one year. 

All 11 U of U Health primary care clinics were eligible for inclusion due to the presence of PPCS pharmacist services at all clinics. Potential provider participants were identified through communication distribution lists. A total of 127 providers were identified as potentially eligible for inclusion in the study.

An e-mail explaining the research and inviting providers to sign up for an interview using a sign-up link was sent to all potentially eligible providers on two separate occasions during the month of December. The e-mail contained language that explained the purpose of the study, outlined the inclusion and exclusion criteria, and assured anonymity. Interviews were conducted throughout the month of January. PPCS pharmacists and technicians also advertised the study to their respective providers in their individual clinics. No incentives were provided for participation. 

The target number of providers was 20 or more from at least four different practice sites to allow for feedback from a variety of perspectives and practice sites. We aimed for four providers from each possible provider credential to allow for feedback from a variety of credentialing backgrounds. For an interview-based study wherein providers are asked to set time aside from their patient schedule, 20 interviews was an ambitious goal. If this was a survey-based project, a lower interview count would have been concerning.

### 2.2. Data Collection and Analysis

Interviews were conducted by one of three pre-identified interviewers, including two pharmacists and one qualitative research expert. The interviewers did not participate in any interview where a prior relationship existed. To accommodate the providers’ limited availability, interviews were conducted virtually over a video call. 

A semi-structured interview template (see Appendix A) was created to allow for follow-up questions and clarification as deemed necessary. The interview template was created with the intent of a neutral presentation for each question. We conducted three pilot interviews. While no other formal validation process was conducted for the interview questions, the responses from the pilot interviews were consistent with all subsequent interviews. Each one-on-one virtual meeting was recorded and transcribed into text by a professional transcription service. To maintain anonymity, the transcribed interviews were labeled with randomly assigned numbers, and any names of pharmacists and technicians used during the interview were replaced with the corresponding individuals’ titles (pharmacist or technician). 

The de-identified transcribed interview transcripts were coded and analyzed for common themes using ATLAS.TI software. Coding was performed independently in duplicate by separate investigators utilizing a systematic approach, fully coding one interview at a time. The two individual coding packets were reconciled to create a final coded transcript. The final coded transcript was reviewed independently in duplicate by each coding investigator for theming via inductive coding. The two independently generated themes were reconciled between the two coding investigators to create the final themes. 

## 3. Results

A total of 25 of 127 eligible providers were interviewed (provider response rate: 19.7%). An additional 12 providers expressed interest in participating via e-mail but could not participate in the predefined time slots (interested response rate: 29.1%). Clinic PPCS pharmacists reported multiple instances where providers expressed verbal interest but were unable to participate in the predefined time slots. The predefined time slots were cited by providers as the primary restriction for participating. In total, 9 of 11 eligible clinics were represented by at least one participating provider (clinic response rate: 81.8%). 

The provider specialties included 17/25 from family practice (68%), 4/25 from geriatrics (16%), 3/25 from internal medicine (12%), and 1/25 from internal medicine/pediatrics. The provider credentials included 19/25 medical doctors (MD), 3/25 physician assistants (PA), 2/25 advanced nurse practitioners (APRN), and 1/25 doctor of osteopathic medicine (DO). 

A total of 17 codes were identified, largely based on the questions asked (see Table 1). Quotations from providers were coded a total of 811 times, with the most frequent codes being “save time” (140), “pharmacists being valuable“ (112), “availability in clinics” (77), and “collaboration” (73). Within all 25 interview transcripts, there were 0 negative and 9 neutral comments (e.g., “I don’t know that my clinical pharmacists really changed how much work I bring home with me. I don’t bring any work home with me because I set that precedent at the beginning of my career”) regarding PPCS pharmacists and technicians. All other comments were considered positive.

Using the coded quotations, four themes were identified (see Table 1). They included “enhanced patient care”, increased job satisfaction”, “reduced workload and burnout”, and “increased PPCS accessibility”.

### 3.1. Enhanced Patient Care

Providers found pharmacists invaluable in helping patients with complex situations and chronic diseases. One provider stated, “We think of our clinical pharmacist as one of our providers. We don’t think of them as a separate entity. We use them as a part of our team. Our patients know them. Our patients ask for them”. Most providers described how pharmacy services provided higher quality and safer patient care. This provider said, “They make me feel confident that I’m providing safe care. So, I feel number one, more confident that I can provide safe care and then efficient care”.

Nearly all providers felt that clinical pharmacists were essential to providing proper evidence-based medicine and that their value far exceeded their cost. Two statements that depict this are “I think the biggest value for the pharmacists is they help me to practice evidence-based medicine. I would not be able to do my job without them. It’s an invaluable resource” and “I don’t think people realize just how valuable and relevant pharmacists are to the delivery of primary care. Being physically present in the practice, participating in the day by day, hour by hour care of our patients. That is hard, if not impossible to replace. And does it cost you? You bet it costs, but it’s worth spending”.

Many described how beneficial clinical pharmacy services were in increasing access to medications through affordability. A provider summarized this by saying, “The ability for the technician to be able to check and see what medications would cost for a particular patient is so valuable. So that you’re not sending them out the door with an insanely expensive medication that they’ll never pick up and never use”.

### 3.2. Increased Job Satisfaction

Every provider said that clinical pharmacy services had an impact on whether they would stay or leave the organization. Some considered it a small factor, with the majority of physicians categorizing it as a major factor. Most providers stated they would not work somewhere where a pharmacist was not a part of the care team. One provider noted, “I just can’t imagine working in any clinical setting without a clinical pharmacist. I would resist anyone’s attempt to try and scale back for financial reasons or anything like that”. Another provider further emphasized, “Once people start working with the pharmacy team, they don’t look back. Once they do experience it and recognize it, there’s really no turning back”.

Providers found the collaboration between pharmacists and themselves to be beneficial. Every provider expressed that having pharmacists as part of their care team has made them happier at work. A provider described this by saying, “I was really, really upset when our previous pharmacist left. I kept doing my job, but I enjoy my work more with a pharmacist. And again, if I feel I’m delivering higher quality care to my patients, that makes me happier at work. Having a pharmacist again has been very much a job satisfier for me and my peers”. A provider at another clinic felt that “Having that extra expertise available is comforting. It’s nice having that extra set of eyes, that other brain working on things with you”.

Many providers felt that pharmacists added to their medical students’ and residents’ training experience. They also expressed how appreciative the learners were to work with pharmacists in their training. This was illustrated by an attending physician who said, “From my attending role, being able to have a pharmacist sitting in the room with us as we’re reviewing patients and talking about stuff is such an amazing resource. We probably turn to the pharmacist at least half a dozen times, if not more, throughout a morning session to ask them questions and recommendations on things that we want to do”.

### 3.3. Reduced Workload and Burnout

Every provider expressed how PPCS saved them time, and many stated that the services allowed them to see more patients and perform more non-medication-related services. One provider said, “I think it takes some of the clinical workload off for me so I can be more efficient when I’m there. So I think it definitely improves how much work I take home”. Another provider stated, “to not having them, I think we’d be suffering a bit. We’d be in pain”.

Every provider also felt that clinical pharmacy services significantly reduced their workloads, with some expressing how their practices are dependent on the service. Two statements that depict this sentiment are “We’ve gotten used to having them there and it’s just the new normal. If we ever had to go back” and “My workload is adapted around the fact that I have a pharmacist. Take the pharmacist away and see what happens, and a year later when all your doctors quit, then you have your answer”. 

Several providers spoke to the fact that PPCS helped to reduce the amount of burnout they feel. One provider recounted their experience with some of their colleagues and burnout by saying, “I have friends across the country that have left Primary Care. One of the reasons why is, they’re having to do all of this team-based care themselves. It’s definitely one of those things that makes burnout much less”.

### 3.4. Increased PPCS Accessibility

Providers consistently expressed a desire for more availability of clinical pharmacy services in their clinics. One quotation that demonstrates this is “We have far less pharmacy support than we need. The places where we have no pharmacy support, the providers are slugging away in kind of the late 20th century model. They don’t have the benefit of that, and we could really benefit by much more support”. Another statement that encapsulates this is “I think that if we had more pharmacists or if the pharmacist had more time, then I think that it would be more valuable to me”. 

Some providers advocated for creating more positions for pharmacists to increase their availability and allow for more utilization of their services, with one stating, “I honestly don’t think that I use them as much as I could because I want to save their sanity because I really like them as people. And I think they are really good. So I don’t end up using them as much as I think I could”. Another provider said, “I think that if we had more pharmacists or if the pharmacist had more time, then I think that it would be more valuable to me”.

Increased accessibility was the only suggestion given by providers for how PPCS could be of greater value to them and their patients. One of the many related comments was “I really appreciate having the pharmacist there, so whatever we could do to keep them there and through their presence in our clinics, I think is going to be best for our providers and the patients”. Yet another provider said, “Having them here in person makes it very easy to curbside or have a discussion or run over and just ask them a quick question. They can come in and see my patient either together or before I see them, and I think that’s really useful. So just having them there in clinic in person, I think is really the biggest thing for me”.

Finally, providers often discussed how increasing access to PPCS services would improve retention within the organization, with one provider stating, “The access to clinical pharmacists really adds to the value of working here. I think the loss of that would be devastating at a time when like we’re already losing providers. So I think it’s really important to protect. And I think as a provider I would be open to helping get the pharmacists’ time reimbursed. They bring initiatives to patients and provide patient care, and their knowledge is really, really important”.

## 4. Discussion

Embedded clinical pharmacy services are becoming more common as healthcare evolves into a more team-based model. As these services, such as PPCS, become more commonplace, understanding what providers value most from them becomes increasingly important. Through a qualitative research methodology, this study was able to provide insight into what providers value most from PPCS utilizing a CMM framework. The identified themes included increased job satisfaction, enhanced patient care, reduced workload and burnout, and a desire for increased access to clinical pharmacy services. 

The robust provider response, coupled with the consistent feedback obtained in the interviews, demonstrates how impactful PPCS are to primary care providers and how these clinical pharmacy services help to fulfill their needs. One surprising result of this study was that there was not a single negative comment on PPCS, and there were only nine neutral comments. This demonstrates how valued these services are to the providers that utilize them. Additionally, increased access to PPCS being the only suggested improvement supports the idea that providers value these services and advocate for additional support for existing clinical pharmacists and technicians. 

Our findings compliment the results of a prior study on a similar topic [14]. Funk et al. found that pharmacists providing comprehensive medication management led to decreased workloads, satisfaction that patients were receiving better care, reassurance, decreased mental exhaustion, enhanced professional learning, increased provider access, and the achievement of quality measures. Each of these outcomes was mentioned multiple times throughout our provider interviews, which helped illustrate the consistent benefit that providers gain from PPCS.

Clinic-embedded pharmacists across the nation must utilize multiple strategies to justify their positions. Currently, there is a general lack of billing options for ambulatory clinical pharmacy services, and as a result, a deficiency in methods to generate revenue [15]. This article highlights a tactic utilized by U of U Health to provide a justification for clinic-embedded pharmacy services by systematically evaluating the providers’ perception of the pharmacists’ value. While the clinical benefit of these services has long-been established, there is less evidence on the providers’ perception of this value using tactics such as the qualitative analysis of interviews and thematic coding [4,5,6,7,8,9,10,11,12,13,14]. While survey studies have been completed, interview-based studies are notably missing from the literature.

Potential confounders and biases were minimized in several ways. First, all materials for recruitment, interviewing, and data analysis were reviewed in duplicate, with the qualitative research expert being one of the reviewers. Second, all interviews were conducted by individuals who had no prior affiliation with the interviewee. This helped to increase the interviewee’s comfort in providing potential negative feedback. Finally, no incentives were provided for participation in the study, which helped ensure that all feedback that was received was due to an authentic desire to help improve PPCS.

### Limitations

One limitation of our study is the potential for response bias. It is possible that those who chose to participate in the interviews did so because they already had a positive perspective on PPCS. This was limited by accepting interviews on a first come first serve basis, and additional time slots and arrangements were not made for providers who reached out for an interview after the slots were full. Another potential limitation was the inclusion of pharmacy-affiliated interviewers. While the potential confounding from this was limited by ensuring the interviewer had no prior relationship with the interviewee, it is still possible that the interviewee felt obliged to share positive comments since the study was run by pharmacy services. A third possible limitation of the study is the limited amount of demographic data that were collected on each interviewee. While this may be seen as a limitation, this was a qualitative study that primarily focused on uncovering and conveying providers’ perceptions of clinical pharmacy services, and more detailed demographic data would not have aided in accomplishing this primary aim. Finally, generalizability is limited due to all interviewees coming from a single healthcare system with a long history of primary care pharmacy. However, while the generalizability is somewhat limited, the extensive history of primary care pharmacy allowed for provider perspectives with varying lengths of exposure to the service, giving a fuller picture of their perceptions. 

## 5. Conclusions

Providers unilaterally expressed that PPCS were of significant value to them through increasing job satisfaction, enhancing patient care, and reducing workloads and burnout. Providers expressed a desire for increased access to PPCS in their clinical practices. The robust provider response and the uniformity of the responses demonstrate the powerful perceived value of and strong desire for increased primary care clinical pharmacy services among modern providers. However, future research is needed to increase the generalizability of these findings. 

## Figures and Tables

**Table 1:** Identified themes and codes with respective frequencies.

Theme/Code	Number of Times Coded (n = 811), % (No.)	Number of Providers with Code (n = 25), % (No.)
**Enhanced Patient Care**	**38.5 (312)**	**100 (25)**
Pharmacists Being Valuable	13.8 (112)	100 (25)
Collaboration	9.0 (73	84 (21)
Diabetes	6.9 (56)	92 (23)
Medication Cost/Coverage	3.9 (32)	64 (16)
Managing Chronic Conditions	2.5 (20)	40 (10)
Continuity of Care	1.2 (10)	32 (8)
Cost of a Pharmacist	1.1 (9)	20 (5)
**Increased Job Satisfaction**	**34.9 (283)**	**100 (25)**
Save Time	17.3 (140)	100 (25)
Stay or Leave the Organization	5.7 (46)	100 (25)
Happiness at Work	4.7 (38)	100 (25)
Integrate into Teams	4.2 (34)	88 (22)
Benefit to Trainees	3.1 (25)	48 (12)
**Reduced Workload and Burnout**	**14.9 (121)**	**100 (25)**
Reduced Workload	6.7 (54)	100 (25)
Burnout	4.3 (35)	96 (24)
Not Taking Work Home/Staying Late	3.9 (32)	96 (24)
**Increased PPCS Accessibility**	**11.7 (95)**	**100 (25)**
Availability of Pharmacists in Clinic	9.5 (77)	96 (24)
In-Person Pharmacists	2.2 (18)	44 (11)

## Data Availability

All data for the study are included within the manuscript.

## Appendix A

Interview Template

1. We would like to collect some basic demographic information.
  - What kind of provider are you? (MD, PA, APRN)
  - What is your specialty
  - What is your clinic location (or locations)?
  - How long have you been practicing medicine?
  - How long have you been working with your embedded clinical pharmacy team?
2. What has your experience been like working with an embedded clinical pharmacy team?
3. Do you find it valuable to work with your PPCS pharmacy team?
  - Tell me more about that.
  - What do you value most?
4. How has your PPCS pharmacy team affected your workload?
  - Can you give an example of this?
5. In what ways has your PPCS pharmacy team affected your happiness at work?
6. In what ways has your PPCS pharmacy team affected your ability to not bring work home with you?
7. How has your PPCS pharmacy team affected your desire to stay with the University of Utah Health?
  - Can you give an example of this?
8. In what ways has PPCS affected you in relation to “burnout”?
9. How could PPCS Pharmacy services provide greater value to you?
10. How could PPCS pharmacy services better integrate into your clinical team?

Thank you for participating in this interview, your feedback is greatly appreciated.

## Appendix B

List of abbreviations

1. CMM—Comprehensive Medication Management
2. PPCS—Pharmacy Primary Care Services
3. CPA—Collaborative Practice Agreement
4. MD—Medical Doctor
5. PA—Physician Assistant
6. APRN—Advanced Nurse Practitioner
7. DO—Doctor of Osteopathic Medicine

## References

[1] The Patient Care Process for Delivering Comprehensive Medication Management (CMM): Optimizing Medication Use in Patient-Centered, Team-Based Care Settings. CMM in Primary Care Research Team. https://www.accp.com/docs/positions/misc/CMM_Care_Process.pdf.

[2] McInnis T., Webb E., Strand L. (2012). Patient Centered Primary Care Collaborative (PCPCC). The Patient Centered Medical Home: Integrating Comprehensive Medication Management to Optimize Patient Outcomes Resource Guide.

[3] Bodenheimer T., Sinsky C. (2014). From triple to quadruple aim: Care of the patient requires care of the provider. Ann. Fam. Med..

[4] Brummel A., Carlson A.M. (2016). Comprehensive Medication Management and Medication Adherence for Chronic Conditions. JMCP.

[5] Isetts B.J., Brown L.M., Schondelmeyer S.W., Lenarz L.A. (2003). Quality assessment of a collaborative approach for decreasing drug-related morbidity and achieving therapeutic goals. Arch. Intern. Med..

[6] Rao D., Gilbert A., Strand L.M., Cipolle R.J. (2007). Drug therapy problems found in ambulatory patient populations in Minnesota and South Australia. Pharm. World Sci..

[7] Isetts B.J., Schondelmeyer S.W., Artz M.B., Lenarz L.A., Heaton A.H., Wadd W.B., Brown L.M., Cipolle R.J. (2008). Clinical and economic outcomes of medication therapy management services: The Minnesota experience. J. Am. Pharm. Assoc..

[8] de Oliveira D.R., Brummel A.R., Miller D.B. (2010). Medication therapy management: 10 years of experience in a large integrated health care system. JMCP.

[9] Smith M., Giuliano M.R., Starkowski M.P. (2011). In Connecticut: Improving patient medication management in primary care. Health Aff..

[10] Turner K., Buu J., Kuzel M., Van Wagoner E., Berrett G. (2020). Early Implementation of comprehensive medication management within an academic medical center primary care pharmacy team. Innov. Pharm..

[11] Livet M., Blanchard C., Sorensen T.D., McClurg M.R. (2018). An Implementation System for Medication Optimization: Operationalizing Comprehensive Medication Management Delivery in Primary Care. J. Am. Coll. Clin. Pharm..

[12] Pestka D.L., Frail C.K., Sorge L.A., Funk K.A., Janke K.K., McClurg M.T.R., Sorensen T.D. (2019). Development of the comprehensive medication management practice management assessment tool: A resource to assess and prioritize areas for practice improvement. J. Am. Coll. Clin. Pharm..

[13] Pestka D.L., Frail C.K., Sorge L.A., Funk K.A., McClurg M.T.R., Sorensen T.D. (2019). The practice management components needed to support comprehensive medication management in primary care clinics. J. Am. Coll. Clin. Pharm..

[14] Funk K.A., Pestka D.L., Roth McClurg M.T., Carroll J.K., Sorensen T.D. (2019). Primary Care Providers Believe That Comprehensive Medication Management Improves Their Work-Life. J. Am. Board. Fam. Med..

[15] Kliethermes M. (2019). Ambulatory pharmacists and their responsibilities in New Healthcare Models. Building a Successful Ambulatory Care Practice.

